# In vivo confocal microscopy of an apparent deep stroma corneal dystrophy: a case report

**DOI:** 10.1186/1757-1626-2-9317

**Published:** 2009-12-14

**Authors:** Michele Lanza, Maria Borrelli, Enrico Benusiglio, Nicola Rosa

**Affiliations:** 1 Department of Ophthalmology, Second University of Naples Via Pansini 5, Naples 80100 Italy; 2 Centro Grandi Apparecchiature, Second University of Naples, Sezione di Ricerca Clinica in Oftalmologia, Via De Crecchio, 16 Naples, 80100 Italy

## Abstract

A 41-year-old white woman was referred to our Department to rule out the presence of a Fuch's corneal dystrophy. On slit-lamp biomicroscopy, small bilateral punctuate opacities appearing mostly in the posterior stroma were observed, suggesting a differential diagnosis of pre-Descemet's dystrophy as opposed to Cornea Farinata.

Confocal microscopy in the central cornea of both eyes revealed the normal appearance of superficial and basal epithelial layers. However throughout the full thickness of the cornea fine highly refractive granules, localized both in the keratocytes cytoplasm and in the stroma matrix were noted. In both eyes abnormal polymegatism and pleomorphism was observed.

## Introduction

Several corneal dystrophies such as Cornea Farinata, Punctuate Dystrophy, pre-Descement's Dystrophy, Fleck's Dystrophy, Deep Filiform Dystrophy and Posterior Punctiform Dystrophy have the clinical appearance of fine opacities limited to the posterior corneal stroma. These deposits are not associated with ocular or systemic diseases, they often are asymptomatic and only very rarely they interfere with visual acuity [1].

Sometimes the differential diagnosis between these dystrophies may prove challenging [1]. In vivo confocal microscopy (CM) has been useful in differentiating several cases and might help to bring about a better classification and discover new entities [2]. In this paper we report a case where (CM) appeared crucial for carrying out diagnosis.

## Case presentation

A 41-year old white woman was referred to us to rule out the presence of a Fuch's corneal dystrophy.

**Figure 1:**
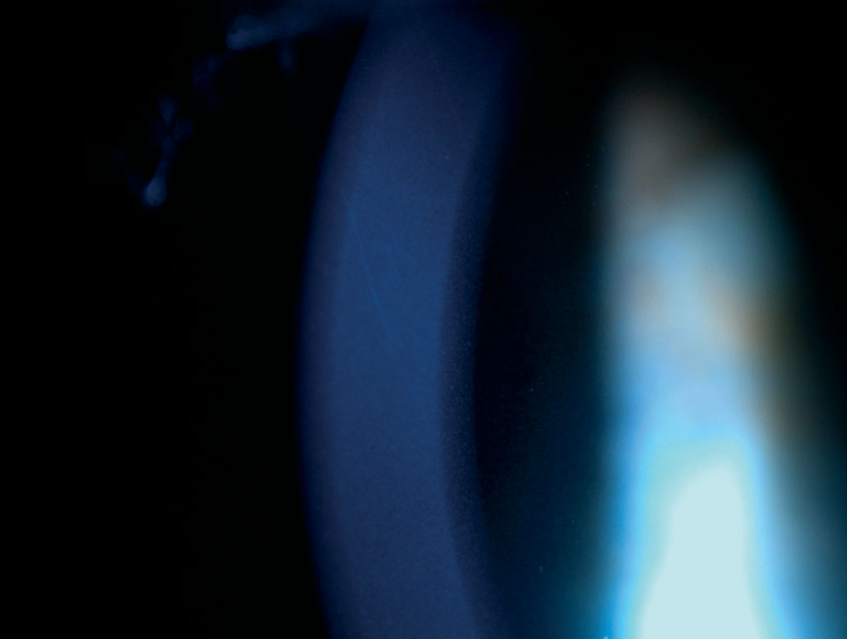
**Slit-lamp photography demonstrating the corneal changes**.

Her family history did not reveal the presence of corneal dystrophies; in both eyes her best corrected visual acuity was 20/20, while intraocular pressure was within normal limits, and the findings on external examination and dilated pupil fundus examination were unremarkable. On slit lamp biomicroscopy small bilateral punctuate opacities mostly located in the posterior stroma were observed, suggesting the differential diagnosis between pre-Descemet's dystrophy as against Cornea Farinata (Figure 1).

The patient underwent in vivo CM with Confoscan 3 (Nidek Technologies, Vigonza, Italy) in order to obtain further details of her corneal disorder.

**Figure 2:**
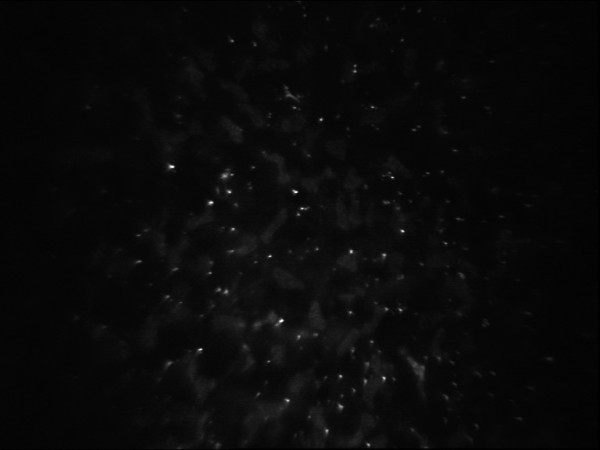
**In vivo confocal microscopic findings of the right eye showing hyperreflective dot-like deposits in the anterior corneal stroma**.

**Figure 3:**
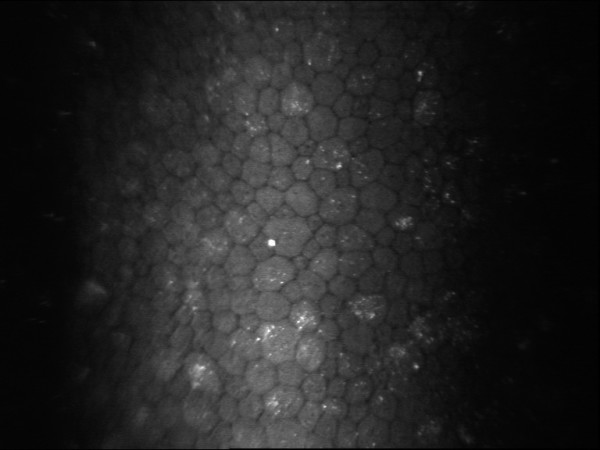
**In vivo confocal microscopic findings of the left eye showing hyperreflective dot-like deposits in the deep stroma adjacent to the corneal endothelium**.

In both eyes it revealed a normal appearance of the superficial and basal epithelial layers. Nevertheless throughout the full thickness of the corneal stroma fine highly refractive granules, localized both in the keratocytes cytoplasm and in the stromal matrix, were observed (Figure 2 and Figure 3), while normal keratocytic nuclei with a typical coffee-bean like appearance were noted. In the right eye the corneal endothelial cell count was 2,512 cell/mm^2 ^with abnormal polymegatism (46.1%) and pleomorphism (37.9%), whereas in the left eye the endothelial cell count was 2,984 cell/mm^2 ^with abnormal polymegatism (49.9%) and pleomorphism (26.9%).

## Discussion

Corneal dystrophies affecting the entire corneal stroma, and in particular the posterior stroma, can often show a similar appearance on slit-lamp examination, thus the differential diagnosis may prove challenging and some of these dystrophies may actually be a variation of the same disease [1].

In vivo CM has been used by several authors in an attempt to clarify the diagnosis of several deep stroma corneal dystrophies.

Cornea Farinata is an asymptomatic degenerative condition characterized by a myriad of fine, dust-like opacities found bilaterally in the posterior stroma near Descemet's membrane. They are best seen on retro illumination and appear grey-brown to white colour. On CM the superficial (dark and light cells) and the mid stroma layers appear normal; highly reflective small particles can be found in the cytoplasm of some keratocytes only in the deep stroma adjacent to the corneal endothelial layer with no abnormalities of the Descemet's membrane and endothelial cell layers [1].

Pre-Descemet's dystrophy, a condition bearing a close clinical resemblance to Cornea Farinata, on slit-lamp examination shows larger and more polymorphous opacities than those of Cornea Farinata, distributed throughout the cornea but with a predilection for the deep stroma [2]. CM demonstrates intra- and extra-cellular hyper-reflective inclusions immediately anterior to Descemet's membrane and prominent sub-basal nerves; the epithelium, basement membrane and endothelium appear normal [3,4].

Fleck's dystrophy is a rare dominant autosomal corneal dystrophy with discrete opacities similar to those of Cornea Farinata and pre-Descemet dystrophy, but the opacities are located not only in the posterior but throughout the entire corneal stroma [4]; intracellular hyper-reflective dots of various shapes throughout the corneal stroma have been reported on CM. Some authors have described the involvement of sub-basal nerves characterized by multiple inclusions similar to those found in the keratocytes [4] while others have suggested that the presumed nerves and inclusions actually represent abnormal keratocytes processes (5).

Deep Filiform dystrophy of Maeder and Danis consists of multiple filiform gray opacities in the pre-Descemet area that affect the entire width of the cornea except for the perilimbal region. The original case occurred in a middle-aged woman with keratoconus. This disorder may represent a degeneration rather than a dystrophy [5].

In Posterior Punctiform dystrophy punctiform elements are present in small groups of filament some of which are of a dendritic type, whereas others resemble canes or comas, and are of a bluish-white colour, being visible to direct and indirect illumination with ring or strictly axial distribution of the opacities [5].

According to our findings our case report does not seem to belong to any of the above described corneal dystrophies both on account of the appearance and distribution of the deposits.

On the basis of our findings we suggest that the classification of these dystrophies should be revised. The diagnosis of pre-Descemet's dystrophies should be limited to those dystrophies where CM rules out the involvement of the anterior and mid-stroma, whereas those with an involvement of the anterior and mid stroma, should be classified differently.

This CM based classification, could be very important for future genetic studies on corneal dystrophies. Clearly, further longitudinal studies on a large number of patients should be performed in order to rule out the possibility that these dystrophies are not evolving pictures of a phenotypic variation of a unique dystrophy, as happened in the so-called Avellino dystrophy case [6].

## Consent

Written informed consent was obtained from the patient for publication of this case report and accompanying images. A copy of the informed consent is available for review by the Editor-in-Chief of this journal.

## Competing interests

The authors declare that they have no competing interests.

## Authors' contributions

LM has been involved in drafting the manuscript and has given final approval of the version to be published BM. has been involved in drafting the manuscript and has given final approval of the version to be published. BE has made substantial contributions to conception and design, acquisition of data and has given final approval of the version to be published.

RN has made substantial contributions to conception and design, acquisition of data and has given final approval of the version to be published
